# Safety, Pharmacokinetics/Pharmacodynamics, and Absolute Bioavailability of Dexmedetomidine Hydrochloride Nasal Spray in Healthy Subjects: A Randomized, Parallel, Escalating Dose Study

**DOI:** 10.3389/fphar.2022.871492

**Published:** 2022-05-20

**Authors:** Yun Kuang, Sai-Ying Wang, Meng-Na Wang, Guo-Ping Yang, Can Guo, Shuang Yang, Xing-Fei Zhang, Xiao-Yan Yang, Qi Pei, Chan Zou, Yan-Hong He, Ying-Yong Zhou, Kai-Ming Duan, Jie Huang

**Affiliations:** ^1^ Center for Clinical Pharmacology, The Third Xiangya Hospital of Central South University, Changsha, China; ^2^ Department of Anesthesiology, Third Xiangya Hospital of Central South University, Changsha, China; ^3^ Research Center of Drug Clinical Evaluation of Central South University, Changsha, China; ^4^ Department of Pharmacy, The Third Xiangya Hospital of Central South University, Changsha, China

**Keywords:** dexmedetomidine nasal spray, pharmacokinetics, pharmacodynamics, absolute bioavailability, healthy subjects

## Abstract

**Background:** The present study evaluated the safety, pharmacokinetics/pharmacodynamics (PK/PD), and absolute bioavailability (Fabs) of Dex nasal spray in healthy adult subjects, which serves as a bridge for the subsequent study in children.

**Methods:** Part 1: a double-blind, placebo-controlled, single ascending dose study was performed on 48 subjects. For 20-/40-μg groups, every 6/2 subjects received either Dex/placebo nasal spray or Dex/placebo injection in two periods. In total, 12/4 subjects each received 100 μg Dex/placebo nasal spray. Part 2: a randomized, double-blind, placebo-controlled study; 12/4 subjects received 150 μg Dex/placebo nasal spray. Part 3: a randomized, open, self-crossover study; 12 subjects received 20 μg and 100 μg Dex nasal spray in two periods alternately. The method of administration was optimized in Part 2 and Part 3.

**Results:** In part 1, Dex nasal spray was well tolerated up to the maximum dose of 100 μg, whereas the Fabs was tolerated to only 28.9%–32.3%. In Part 2 and Part 3, the optimized nasal spray method was adopted to promote the Fabs of Dex nasal spray to 74.1%–89.0%. A severe adverse event was found in Part 2. In Part 3 (100 μg), the Ramsay score increased the most and lasted the longest, whereas the BIS score decreased most significantly.

**Conclusion:** Using the optimized nasal spray method, a single dose of 20/100 μg of the test drug was safe and tolerable, and 100 μg may have approached or reached the plateau of sedation. In addition, it is found that the optimized method can greatly improve the bioavailability of the test drug, leading to its higher reference value.

## 1 Introduction

It has been well established that children are not small adults, but rather they are a distinct and heterogeneous patient group (Ivanovska et al., 2014; Wimmer et al., 2015). However, more than 50% of drug formulations are not age-appropriate for most of the pediatric groups (delMoral-Sanchez et al., 2020; Ye et al., 2013). Nowadays, formulation research and development in the pediatric area remains essential (Smyth et al., 2012). Sedation drugs are usually required to reduce or eliminate adverse events (AEs) caused by anxiety and panic caused during the diagnosis and treatment process in pediatric patients. However, there is no unique sedative drug specifically for children with a good sedative effect, high safety, and convenience of administration.

Dexmedetomidine (Dex), a selective α_2_ adrenergic receptor agonist with remarkable sedative and hypnotic effects, was initially approved for i.v. sedation in intensive care units by the FDA in 1999 (Cui et al., 2014). Based on the small dosage needed, easy awakening, and absence of respiratory depression, the FDA further extended the indicators of non-intubated patients prior to surgery and other procedures and/or during surgery in 2008, (Weerink et al., 2017) as Dex has a unique synergistic effect on the physiological and psychological needs of critically ill patients, significantly reducing the dose for anesthesia induction (Phan and Nahata, 2008). The National Medical Products Administration (NMPA) approved generic Dex produced by Hengrui Medicine Co., Ltd. in 2009 (Aibeining).

The intranasal route of medication administration is becoming increasingly popular in patients without intravenous access or in those in whom i.v. is difficult (children or uncooperative adults) (Rech et al., 2017). At present, there is no dosage form of Dex specifically designed for intranasal administration in the market. However, the off-label use of Dex injection administered by drops from a syringe or by nasal mucosal atomization has been frequently reported in the literature (Li et al., 2018). Many studies have confirmed that intranasal Dex can achieve satisfactory sedation and acceptable safety (Jun et al., 2017; Kim et al., 2017; Tervonen et al., 20201992). But, when unmodified intravenous preparations are used for intranasal administration, the dosage cannot be accurately controlled, resulting in huge differences in bioavailability (Iirola et al., 2011). Therefore, there is an urgent need for a stable Dex nasal spray to solve the current issue.

Jiangsu Hengrui Medicine Co., Ltd., developed dexmedetomidine nasal spray (chemical classification of NMPA, category 2), which is expected to solve the aforementioned problems. This nasal spray does not require drug configuration and can be used directly, with two fixed doses: 10µg/50µl/spray per spray and 25µg/50µl/spray per spray (the active ingredient content of each spray is within 80%–120% of the labeled amount and no less than eight sprays per bottle), which can accurately control the dose.

Because of ethical issues and recruiting difficulties, rare studies were performed directly on children in China. Individual differences in intranasal administration of dexmedetomidine are not known, so pharmacokinetics/pharmacodynamics (PK/PD) data of nasal spray administration in adults were needed first. The bridge was then connected to the PK/PD study in children. This study was conducted to evaluate the safety, PK/PD, and absolute bioavailability (Fabs) of Dex nasal spray in healthy adult subjects, which will serve as a bridge for subsequent research in children.

## 2 Materials and Methods

### Subjects

Healthy Chinese subjects aged 18–40 years with a body mass index (BMI) in the range of 19.0–26 kg/m^2^ were eligible for inclusion. They were ascertained to be healthy after a medical interview, physical examination, clinical laboratory tests, 12-lead electrocardiogram (ECG), and vital sign measurement. Patients with systolic blood pressure <90 mmHg or >140 mmHg, diastolic blood pressure (DBP) < 50 mmHg or >90 mmHg, and heart rate (HR) < 50 bpm or >100 bpm were excluded during the screening period. Nonetheless, patients not suitable for nasal spray administration or unwilling to receive nasal administration and patients with abnormalities in thyroid function, blood oxygen saturation, and other situations were excluded from the study. However, in Part 3, the lower limit of DBP and HR in the exclusion criteria was raised (DBP <60 mmHg or >90 mmHg; HR < 60 bpm or >100 bpm).

The study was reviewed and approved by the Ethics Committee of The Third Xiangya Hospital, Central South University (ethical approval numbers: 2016L09138, 2018B02196, and 2018B02197). The study was conducted according to the Declaration of Helsinki and Good Clinical Practice (GCP) guidelines. All subjects gave written informed consent before any study-related procedures were performed. The study was registered with the China Clinical Trials Registry (numbers: ChiCTR-IIR-17013180 and ChiCTR1900026141).

### Study Design

#### Part 1

This study used a randomized, double-blind, placebo-controlled, single ascending dose design to evaluate the safety, PK/PD, and Fabs of Dex nasal spray in healthy Chinese subjects. Three dose levels, each with 16 patients, were planned as follows: 20, 40, and 100 μg (groups A, B, and C). The starting dose was based on the NOAEL from a 4-week toxicity test performed in immature rats. The 20- and 40-μg groups were subjected to a two-period, two-treatment crossover design, while the 100-μg group was subjected to a one-period design. In the 20-/40-μg groups, every 6/2 subjects received either Dex/placebo nasal spray or Dex/placebo injection in the first period and switched over to the other treatment after a washout of 7 days. In the 100-μg group, 12/4 subjects only received Dex/placebo nasal spray. Escalation to the next dose level proceeded only when the current dose demonstrated good tolerability.

Dex/placebo Injection was diluted with 20 or 40 ml of 0.9% saline and infused at a constant rate for 15 min using an intravenous infusion pump. For the Dex/placebo nasal spray, the nostrils of the patients were cleaned, by making them sit with a straight upper back and by tilting their heads forward at an angle of 5–10°. The number of sprays (dose/drug specification) was calculated according to the protocol designed, and the nozzle was inserted into the nasal cavity, which was diagonally facing toward the outer corner of the eye and not the nasal septum, and then the drug was sprayed evenly into the left and right nostrils. After finishing all sprays, the head was slightly tilted back at an angle of 20–30°, and the formulation was inhaled slowly for about 10 s, then the supine position was assumed. For example, the 100-µg group needed four sprays (100 µg/25 µg*spray^−1^), for which the drug was sprayed into both nostrils in the order of left-right-left-right-head up and then inhaled for 10 s.

#### Part 2

Part 2 is based on the recommended dose of Dex injection (1 μg/kg, 60 kg) and the Fabs of Dex nasal spray in Part 1 (about 30%). The recommended dose of Dex injection for adults is 60 µg (1 μg/kg, 60 kg). To achieve an exposure equivalent to injections, about 200 µg nasal spray is needed. In addition, a loss of drugs was found during the previous procedure, and the nasal spray administration method has been optimized according to the recommendations of experienced clinicians. Thus, we have further explored the effect of a 150 μg dose (group D). Using a randomized, double-blind, placebo-controlled trial design, 16 healthy subjects were enrolled, of which 4 subjects were randomized to take a placebo.

In addition, the nasal spray administration method has been optimized according to the recommendations of experienced clinicians. When more than one spray was needed for the same nostril, after giving one spray, the head was tilted back at 20–30° and inhalation was performed for 30 s before the next spray. The aforementioned steps were repeated until all sprays were over. For example, the order for 100-µg groups was: left-head up for 30 s, right-head up for 30 s, left-head up for 30 s, and then right-head up for 30 s.

#### Part 3

The PK results of the 150-μg group showed that the increase in the ratio of C_max_ and AUC was greater than that of the dose in Part 1. The overall incidence of AEs increased, and one case of serious AE (cardiac arrest) occurred in group D. Therefore, group E was reselected with doses of 20 and 100 μg to further verify the PK characteristics through a randomized, open, two-period, self-crossover design. In group E, 12 healthy subjects were enrolled, wherein every six subjects received either 20 μg or 100 μg Dex nasal spray in the first period, and then they crossed over to the other dosage after a washout period of 7 days. The method of administration was the same as in Part 2.

### Study Drug

Test drug: Jiangsu Hengrui Medicine Co., Ltd. supplied Dex hydrochloride nasal spray (1.0 g: 200 μg 10 µg/50 µl/spray, lot 16120911 and lot 18112201, respectively, and 1.0 g: 500 μg, 25 µg/50 µl/spray, lot 18032201). Groups A and B received 10 µg/**50** µl/spray, and groups C, D, and E received 25 µg/50 µl/spray.

Positive reference drug: Dex hydrochloride injection (2 ml: 200 μg, lot 170429BP) was also supplied by Jiangsu Hengrui Medicine Co., Ltd.

Placebo-controlled drug: Corresponding with two administration methods, there are two formulas of placebo, intranasal placebo (lot 161209-1 and lot 18032401), and injection placebo (lot 170520BC).

### Pharmacokinetic Evaluations

Briefly, 4 ml of blood was collected using an EDTA-K2 anticoagulant tube 30 min before the dose (pre-dose; −30 min) and 5, 10, 15, 20, 30 and 45 min, 1, 1.5, 2, 3, 4, 6, 8, and 10 h post-dose. The plasma Dex concentration was measured for evaluation of PK in groups A, B, C, D, and E. In addition to those mentioned previously, three more samples were collected for groups C, D, and E (at 12, 16, and 24 h). The PK samples were centrifuged at 3000 rpm at 4°C for 10 min. Each plasma sample was divided into two aliquots and stored at −80°C until further bioanalysis. The plasma concentrations of Dex were determined using the HPLC–MS/MS method and expressed as the mean and standard deviation (SD) at each time point. The linear range of plasma concentration detection was 2–2000 pg/ml, and the lower limit of quantitation (LLOQ) was 2 pg/ml.

PK analysis was performed using the noncompartmental model, WinNonlin 7.0 (Pharsight Corporation, Mountain View, CA, United States). The actual sampling time of each point was used for analysis. The main PK parameters included the peak concentrations in plasma (C_max_), time of maximum observed drug concentration (t_max_), the area under the plasma concentration–time curve (AUC_0–t_), plasma clearance (CL), terminal half-life (t_1/2_), the elimination rate constant (λ_z_), and Fabs.

### Pharmacodynamic Evaluations

The Ramsay sedation score (RSS) was used to measure the effect of Dex on the coordination of the extraocular muscles, and the bispectral index (BIS) was used to monitor the level of sedation. The RSS/BIS monitoring was performed at 30 min before the dose (pre-dose) (−30 min) and 5, 10, 15, 20, 30, and 45 min and 1, 1.5, 2, and 3 h after the dose (post-dose). The descriptive analysis of the arithmetic mean, SD, median, and maximum and minimum of the measured and changed values of the RSS/BIS at each time point was performed. In addition, the average efficacy–time curve was drawn.

### Safety Evaluations

Safety was assessed according to the incidence and severity of AEs. All AEs that occurred during the clinical study were required to be reported, including subject interviews, abnormalities in vital signs, laboratory examination, and percutaneous oxygen saturation (SpO_2_). The clinical significance was determined by the monitoring physician. If clinical abnormalities were present, further follow-ups were required until the laboratory examination values or vital sign levels of the abnormal items returned to normal values or stable levels. AEs were assessed by close observation, nonspecific inquiry, and AE records, and the clinical significance of abnormal laboratory examination values was determined by physicians to assess safety.

### Statistical Analyses

PK parameters were summarized by the dose group using descriptive statistics, including n, mean ± SD, and median (range). This method was also used to identify the occurrence of AEs.

The power function model was used to analyze the relationship between AUC, C_max_, and the dose of Dex nasal spray if the 95% confidence interval (CI) of β value was 1.000. It was determined that it meets linear pharmacokinetics. The Fabs of intranasally administered Dex was calculated using the 90% CI of the geometric mean ratio (GMR) (nasal spray/injection) of AUC_0-∞_ converted by logarithm.

## 3 Results

### Participants

This study was conducted between July 2017 and December 2019. In total, 48 healthy Chinese subjects were enrolled in Part 1. Of these subjects, 36 were administered 20, 40, or 100 μg of the test drug and 24 were injected with 20 or 40 μg of the positive drug, whereas 12 were administered the placebo. In Part 2, 12/4 healthy Chinese subjects were enrolled and took a 100 μg test drug/placebo. In Part 3, 12 healthy Chinese subjects were enrolled to receive 20 or 100 μg of the test drug in two periods. A total of 76 subjects, half males and half females, completed the study and were included in the outcome analysis. There was no significant difference in age, height, weight, and BMI among all groups (Table 1).

**TABLE 1:** Demographic characteristics of the study subjects.

	Part 1				Part 2		Part 3
	20 μg (n = 12)	40 μg (n = 12)	100 μg (n = 12)	Placebo (n = 12)	150 μg (n = 12)	Placebo (n = 4)	20/100 μg (n = 12)
Male/female (n/n)	6/6	6/6	6/6	6/6	6/6	2/2	6/6
Age (year)	21.5 (19–35)	25.5 (19–38)	21.5 (18–38)	25.5 (18–33)	21.5 (19–32)	20.5 (20–24)	22.5 (18–26)
Height (cm)	164.3 ± 7.7	164.0 ± 6.8	166.6 ± 6.4	162.7 ± 8.2	161.4 ± 7.6	163.4 ± 9.3	165.5 ± 9.9
Weight (kg)	56.9 ± 8.3	57.5 ± 5.2	59.1 ± 6.2	57.7 ± 5.9	58.2 ± 7.6	57.0 ± 1.9	60.6 ± 11.3
BMI (kg/m^2^)	21.0 ± 1.7	21.4 ± 1.8	21.3 ± 2.0	21.8 ± 2.1	22.3 ± 1.9	21.5 ± 2.0	22.0 ± 1.6

Notes: Data are expressed as mean ± SD, except sex (male/female), which is n/n and age (year), which is median (min to max).

Abbreviations: BMI, body mass index; SD, standard deviation.

### Safety Evaluations

In Part 1, 37 AEs were observed in 22 (61.1%) of the 36 subjects who were administered the test drug, 46 AEs were observed in 23 (95.8%) of the 24 subjects who were administered the positive drug, and 20 AEs were reported in 11 (91.7%) of the 12 subjects who were administered the placebo. AEs were identified in the different dose groups: 20 μg (NS, nine subjects, 14 cases; i.v., 12 subjects, 26 cases), 40 μg (NS, six subjects, 12 cases; i.v., 11 subjects, 20 cases), 100 μg (NS, seven subjects, 11 cases), and placebo (NS, 10 subjects, 13 cases; i.v., four subjects, 7 cases). Of these, 14 AEs in 13 subjects may be related to the test drug, whereas 34 AEs in 21 subjects may be related to the positive drug. All AEs in the test group were of grade 1–2 intensity and dose-independent.

In Part 2, 56 AEs occurred in 12 (100%) of the 12 subjects who were administered a single 150 μg dose of the test drug, of which 51 were considered related to treatment. Nonetheless, 5 AEs occurred in 3 of 4 subjects (75.0%) who were administered the placebo. There were 3 AEs of grade 2 severity, all of which were vertigo, and the symptoms disappeared after an oral or IV dose of 10% glucose. There was also 1 case of severe adverse event (SAE), cardiac arrest. Cardiopulmonary resuscitation was given immediately, followed by glucose supplementation (50 ml IV bolus of 10% glucose injection), and IV atropine sulfate, totaling 1.0 mg in divided doses. After being out of danger, he was hospitalized for further observation and treatment until he recovered. Except for these, all other AEs were of grade 1 in severity.

In Part 3, there were 22 AEs in 11 subjects (91.7%) who were administered a single 20 μg dose of the test drug, with 19 AEs related to treatment. Moreover, 33 AEs were found in 11 subjects (91.7%) of the 100-μg group, with 29 AEs related to treatment. A total of 8 cases of grade 2 AEs occurred in this part, of which 1 case of bradycardia and 1 case of hypoxemia occurred in the 20-μg group and 3 cases of hypotension and 2 cases of hypoxemia occurred in the 100-μg group. Bradycardia was relieved by using an atropine sulfate injection. The symptoms of hypotension were relieved or disappeared after giving ephedrine hydrochloride injection, and hypoxemia disappeared after oxygen inhalation.

In summary, the most common drug-related AEs (Adverse Drug Reactions, ADRs) of Dex nasal spray were hypotension, slow respiration, vertigo, and bradycardia. No deaths or discontinuations occurred due to AEs in this study. Details of the ADR are summarized in Table 2.

**TABLE 2:** ADRs of healthy subjects after a single dose of dexmedetomidine nasal spray or injection.

ADR, n (%)	Part 1								Part 2	Part 3		Total		
	20μg NS, n = 12	20 μg i.v., n = 12	40μg NS, n = 12		40 μg i.v., n = 12		100μg NS, n = 12		150μg NS, n = 12	20μg NS, n = 12	100μg NS, n = 12	IV N = 24	NS N = 60	
All ADRs														
Any grade	3 (25.0)	10 (83.3)	4 (33.3)		11 (91.7)		6 (50.0)		12 (100.0)	10 (83.3)	10 (83.3)	21 (87.5)	36 (60.0)	
Grades 1 or 2	3 (25.0)	10 (83.3)	4 (33.3)		11 (91.7)		6 (50.0)		12 (100.0)	10 (83.3)	10 (83.3)	21 (87.5)	36 (60.0)	
Grade 4	0	0	0		0		0		1 (8.3)	0	0	0	1 (1.7)	
Hypotension	0	5 (41.7)	4 (33.3)		9 (75.0)		3 (25.0)		8 (66.7)	4 (33.3)	7 (58.3)	14 (58.3)	23 (38.3)	
ECG abnormality	0	0	0		1 (8.3)		1 (8.3)		0	0	0	1 (4.2)	1 (1.7)	
Bradycardia	0	1 (8.3)	0		0		0		5 (41.7)	2 (16.7)	1 (8.3)	1 (4.2)	7 (11.7)	
Hypoxemia	0	0	0		1 (8.3)		0		1 (8.3)	1 (8.3)	2 (16.7)	1 (4.2)	3 (5.0)	
Slow respiration	0	0	0		0		0		2 (16.7)	6 (50.0)	4 (33.3)	0	9 (15.0)	
High TBA	0	0	0		0		0		0	1 (8.3)	1 (8.3)	0	2 (3.3)	
Elevated ALT	0	0	0		0		0		0	1 (8.3)	0	0	1 (1.7)	
Vasoconstriction	0	5 (41.7)	0		4 (33.3)		0		0	0	0	9 (37.5)	0	
Orthostatic hypotension	0	1 (8.3)	0		0		0		0	0	0	1 (4.2)	0	
Vertigo	2 (16.7)	1 (8.3)	0		1 (8.3)		0		7 (58.3)	0	3 (25.0)	2 (8.3)	12 (20.0)	
Drowsiness	1 (8.3)	0	0		0		0		5 (41.7)	0	1 (8.3)	0	7 (11.7)	
Headache	0	0	0		1 (8.3)		0		1 (8.3)	0	0	1 (4.2)	1 (1.7)	
Syncope	0	0	0		0		1 (8.3)		0	0	0	0	1 (1.7)	
Nausea	0	1 (8.3)	0		0		0		2 (16.7)	0	0	1 (4.2)	2 (3.3)	
Vomiting	0	0	0		0		0		2 (16.7)	0	0	0	2 (3.3)	
Dry mouth	0	1 (8.3)	0		0		0		3 (25.0)	0	2 (16.7)	1 (4.2)	5 (8.3)	
Hyperbilirubinemia	0	1 (8.3)	0		0		0		0	0	0	1 (4.2)	0	
Hyperuricemia	0	1 (8.3)	0		0		1 (8.3)		0	0	0	1 (4.2)	1 (1.7)	
Cardiac arrest	0	0	0		0		0		1^a^(8.3)	0	0	0	1 (1.7)	
Fatigue	0	0	0		0		0		3 (25.0)	0	0	0	3 (5.0)	

a Notes: SAE.

Abbreviations: ADR, adverse reaction (adverse event related to treatment); NS, nasal spray; i.v., intravenous; ECG, electrocardiographic; TBA, total bile acid; ALT, alanine aminotransferase; SAE, serious adverse event.

### Pharmacokinetic Evaluations

**FIGURE 1:**
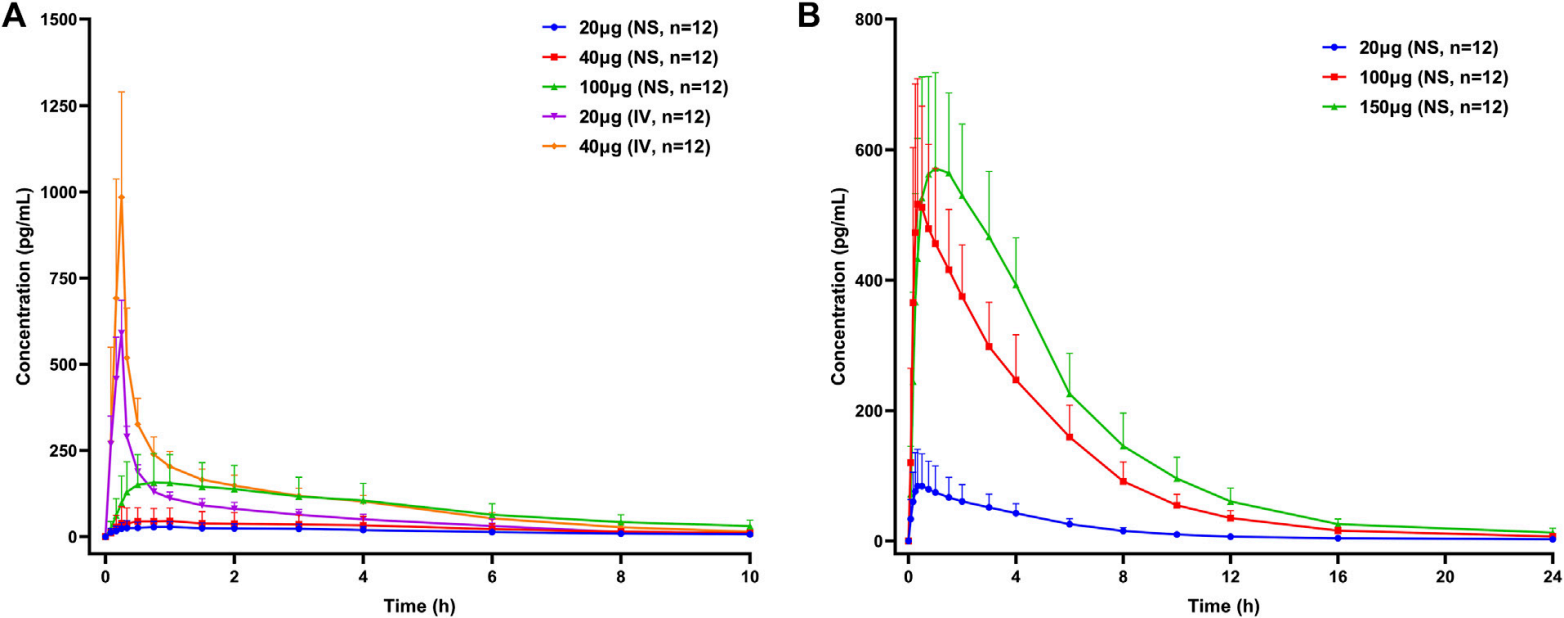
Mean concentration–time curves of dexmedetomidine in healthy subjects following a single dose of dexmedetomidine nasal spray or injection. **(A)** Part 1, **(B)** Part 2, and Part 3. NS, nasal spray; IV, intravenous.

The mean concentration–time curves of Dex are presented in Figure 1. The PK parameters of Dex in the different groups are presented in Table 3.

**TABLE 3:** Pharmacokinetic properties of dexmedetomidine in healthy subjects after a single dose of dexmedetomidine nasal spray or injection.

PK parameter	Part 1					Part 2	Part 3	
	20μg NS, n = 12	20 μg i.v., n = 12	40μg NS, n = 12	40 μg i.v., n = 12	100 μg NS, n = 12	150 μg NS, n = 12	20 μg NS, n = 12	100 μg NS, n = 12
C_max_ (pg/ml)	30.97 ± 19.76	589.92 ± 95.88	50.62 ± 43.47	984.26 ± 305.28	167.36 ± 85.15	611.01 ± 131.94	94.89 ± 49.34	556.25 ± 178.11
AUC_0-t_ (pg*h/mL)	161.76 ± 85.29	600.32 ± 130.14	260.69 ± 193.88	1,070.31 ± 222.80	1,046.47 ± 442.88	3643.77 ± 730.00	410.77 ± 141.81	2,536.82 ± 592.46
AUC_0-∞_ (pg*h/mL)	220.76 ± 96.78	620.34 ± 135.95	367.61 ± 193.93	1,117.45 ± 245.05	1,102.21 ± 444.51	3718.87 ± 728.76	428.99 ± 141.42	2,577.08 ± 597.84
T_max_ (h)	1.00 (0.33–6.00)	0.25 (0.25–0.25)	0.88 (0.25–6.00)	0.25 (0.23–0.28)	0.75 (0.33–2.00)	0.88 (0.34–3.00)	0.50 (0.25–4.01)	0.50 (0.25–2.00)
t_1/2_ (h)	4.33 ± 1.62	2.01 ± 0.34	5.81 ± 3.37	2.14 ± 0.34	5.47 ± 1.92	3.82 ± 0.71	3.42 ± 0.99	4.15 ± 1.04
λ (1/h)	0.18 ± 0.05	0.36 ± 0.06	0.16 ± 0.09	0.33 ± 0.05	0.14 ± 0.05	0.19 ± 0.04	0.22 ± 0.06	0.18 ± 0.04
V/F (×10^5^ml)	7.08 ± 4.71	0.97 ± 0.22	13.45 ± 12.56	1.13 ± 0.19	8.40 ± 4.58	2.31 ± 0.68	2.66 ± 1.48	2.64 ± 1.69
CL/F (×10^5^ ml h^−1^)	1.12 ± 0.58	0.33 ± 0.06	1.36 ± 0.61	0.37 ± 0.07	1.09 ± 0.52	0.42 ± 0.08	0.51 ± 0.16	0.42 ± 0.17
MRT_0-t_ (h)	4.12 ± 0.80	2.25 ± 0.30	4.28 ± 0.77	2.43 ± 0.27	5.97 ± 1.45			
MRT_0-∞_ (h)	6.78 ± 2.64	2.59 ± 0.36	9.09 ± 4.84	2.85 ± 0.39	7.46 ± 2.55			

Notes: Values are presented as mean ± SD, except T_max_, which is the median (min to max).

Abbreviations: PK, pharmacokinetics; NS, nasal spray; i.v., intravenous; Cmax, maximum plasma concentration; AUC_0-t_, area under the concentration curve from 0 time to the last time point; AUC_0-∞_, area under the concentration curve from 0 time to infinity; T_max_, time to C_max_; t_1/2_, terminal elimination half-life; λ, first-order elimination rate constant; V/F, apparent volume of distribution corrected by bioavailability; CL/F, clearance corrected by bioavailability; MRT_0-t_, mean residence time from 0 time to the last time point; MRT_0-∞_, mean residence t.

In Part 1, the Fabs of Dex nasal spray, based on the ratio for AUC_0-∞_ between the nasal spray and IV formulation, following a single dose of 20 and 40 μg, was 32.3% (90% CI, 25.0%–41.8%) and 28.9% (90% CI, 23.1%–36.3%), respectively. Dose proportional analysis showed that the 90% CI of β values of log-transformed C_max_, AUC_0-t_, and AUC_0-∞_ of Dex nasal spray in the range of 20–100 μg were 0.770–1.462, 0.789–1.374, and 0.813–1.232, respectively. All contain 1, indicating linear pharmacokinetics.

Compared with Part 1, the nasal spray administration methods in Part 2 and Part 3 were optimized and kept consistent, so we will discuss the PK/PD of the two parts together. With reference to the PK data of Dex injection in Part 1, the Fabs of 20 μg (group E), 100 μg (group E), and (group D) Dex nasal spray were 74.1%, 89.0 and 85.6%, respectively. Dose proportional analysis of these three doses showed that the 95% CI of β of C_max_, AUC_0-∞_, and AUC_0-t_ was 0.865–1.182, 0.990–1.207, and 1.002–1.226, respectively. The lower limit of “1.002–1.226” is slightly greater than 1. In this case, we still believe that dose-proportionality for C_max_, AUC_0-∞_, and AUC_0-t_ of Dex nasal spray is 20–150 μg.

### Pharmacodynamic Evaluations

Part 1: The BIS scores were reduced after nasal spray, injection, and placebo administration. The BIS was reduced as the dose of Dex increased. The group D at the same dose caused a more significant decrease in BIS than the nasal spray group. The RSS was found to increase after all treatments. There was no significant difference between the nasal spray group and placebo group, but the injection group had a tendency to cause a rapid rise in the RSS.

**FIGURE 2:**
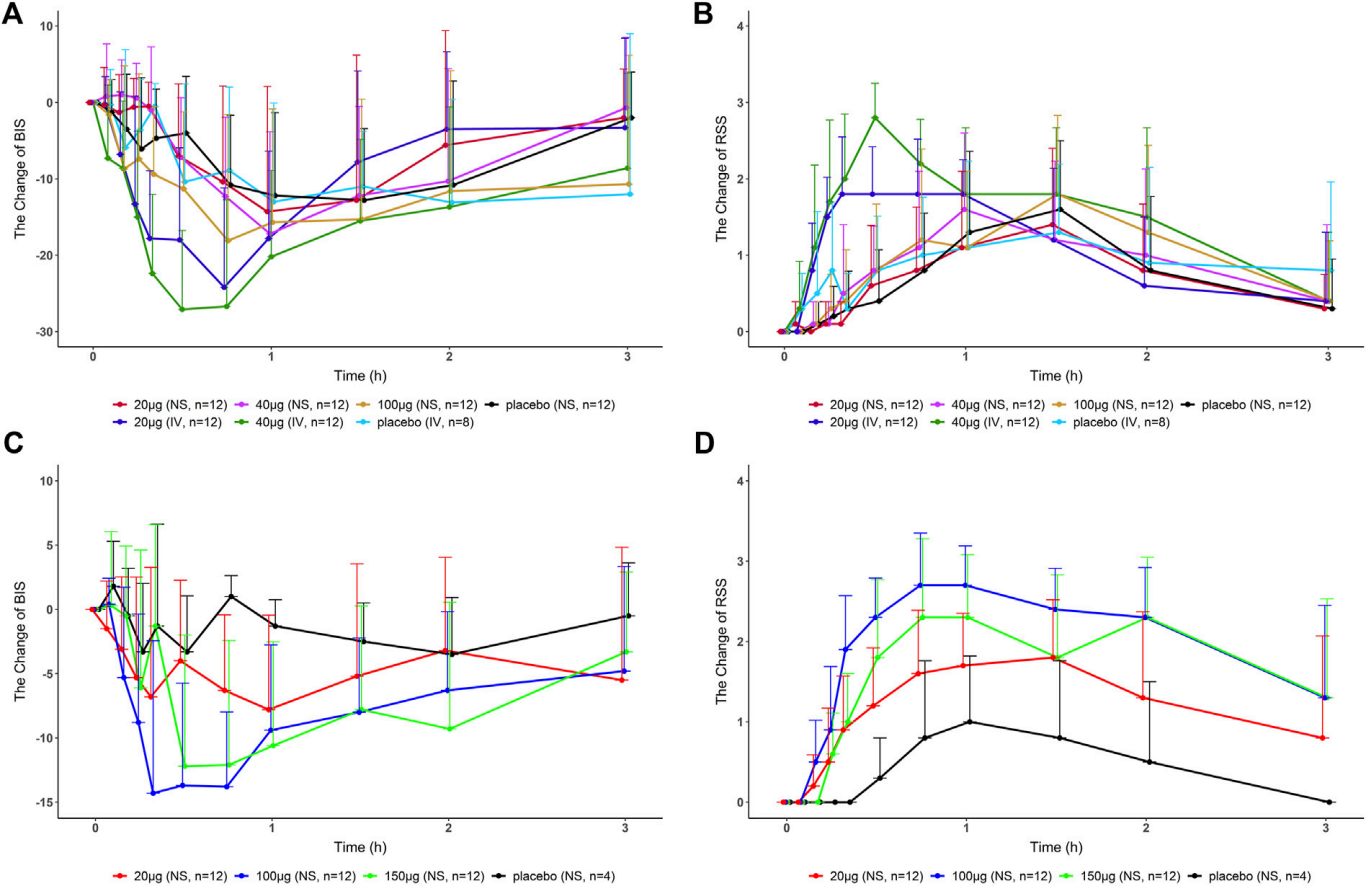
Change in BIS/RSS values compared with the baseline-time curves in healthy subjects after a single dose of dexmedetomidine nasal spray, injection, or placebo. **(A)**, **(B)** Part 1. **(C)**, **(D)** Part 2, and Part 3. BIS, bispectral index; RSS, Ramsay sedation score; NS, nasal spray; IV, intravenous.

Parts 2 and 3: The decrease in BIS in the nasal spray groups (20, 100, and 150 μg) was greater than that of the placebo. Among them, BIS of 100 and 150 μg decreased the most, and there was no significant difference between the two doses. The RSS increase in the nasal spray group was greater than that in the placebo group, and the RSS of 100 μg increased the most and lasted the longest. It suggests that 100 μg may be close to or reach the plateau of sedation (Figure 2).

## Discussion

According to the guidelines on the need for non-clinical testing in juvenile animals on human pharmaceuticals for pediatric indications issued by EMEA/CHMP/SWP (FDA, 2006; EMEA, 2008), immature 22- to 23-day-old rats were selected for a 1-month long-term toxicity test. The lowest effective dose is 10 μg/kg and the NOAEL is 42.61 μg/kg. The safe and effective dose of intranasal Dex for adults and children is 1–2 μg/kg (Wang et al., 2014; Miller et al., 2018; Uusalo et al., 2019). According to the body surface area conversion, we finally chose 20, 40, and 100 μg for this study (Part 1). The results showed that the Fabs of Dex nasal spray was about 30%. The recommended dose of Dex injection for adults is 60 µg (1 μg/kg, 60 kg). To achieve an exposure equivalent to injections, about 200 µg nasal spray is needed. In addition, a loss of drugs was found during the previous procedure, and the nasal spray administration method has been optimized according to the recommendations of experienced clinicians. Thus, we chose 150 μg for further exploration (Part 2). On repeated administrations to the same nostril, the subjects raised their heads for 30 s to ensure that the drug was fully absorbed. The optimized drug delivery method increased the Fabs to about 85%. At the same time, a life-threatening SAE occurred at this dose, which may be related to the excessively high dose and the greatly improved drug absorption. In response to the situation, we used the optimized dosing method to conduct a 20-/100-μg self-control study to verify the PK characteristics (part 3).

Hypotension and bradycardia were also reported to be the most common AEs of Dex injection (Precedex) (Keating, 2015; Weerink et al., 2017; Shehabi et al., 2019). Vertigo, usually accompanied by hypotension or lower heart rate, mainly occurred in the 150-μg group, which may be related to the early getting out of bed after waking up (3–4 h after Dex) because the blood drug concentration was still high (about 1/2 C_max_). Although slow respiration was more common, its incidence was not dose-dependent, and the placebo group also had an incidence of 25.0%. In addition, the respiratory rate remained above 10 beats per minute (bpm), which did not reach the degree of respiratory depression (<8 bpm). Cardiac arrest, an SAE, occurred in the 150-μg group. The main reasons were as follows: first of all, drug factors: Dex injection has been reported to cause clinically significant bradycardia and sinus arrest when there is high vagal tone or different administration methods are used (Zhang et al., 2010; Yeoh et al., 2018). Second, individual factors: this subject’s HR was low before the administration (50–55 bpm) and was fluctuating at 44–53 bpm within 3 h after administration. Third, other factors: there was still a high drug concentration when the subject woke up and got out of bed. In addition, orthostatic hypotension can easily occur after prolonged bed rest, but due to sympathetic nerve inhibition, the slower HR cannot compensate for blood supply, leading to syncope and even cardiac arrest (Ichinose and Nishiyasu, 2012; Iwase et al., 2014).

**FIGURE 3:**
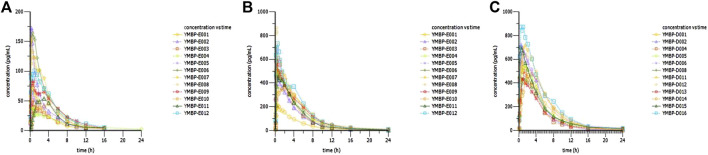
Concentration–time curves of dexmedetomidine in healthy subjects following a single dose of dexmedetomidine nasal spray. **(A)** 20 μg in Part 3. **(B)** 100 μg in Part 3. **(C)** 150 μg in Part 2.

According to the difference in administration, Part 1 was analyzed separately, and Part 2 and Part 3 were analyzed together in the PK/PD part. After optimizing the method of administration, the Fabs of Dex nasal spray increased from 30% to 85%, which means that when the subjects slightly tilted their heads back and inhaled for 30 s between two sprays with the same nostril, an improvement in drug absorption and bioavailability was observed. The Fabs of Dex nasal spray is greater than that of Dex injection for nasal drops (about 65%) (Iirola et al., 2011), which is consistent with the theory (Daley-Yates and Baker, 2001; Gasthuys et al., 2020). Compared with Part 1, the inter-individual variability (RSD%) of AUC and C_max_ in parts 2 and 3 was significantly reduced (19.6%–34.5% and 21.6%–52.0% vs. 40.3%–74.4% and 50.9%–85.9%), which was comparable to injections (20.8%–21.9% and 16.3%–31.0%). This phenomenon has also indicated that a well-optimized intranasal delivery system would most likely reduce inter-individual variability (Iirola et al., 2011). The nasal spray after the optimized administration method is more comparable to the injection (Table 4). Compared with the injection group (20 and 40 μg), the T_max_ of nasal spray (parts 2 and 3) was slightly delayed (0.25 vs. 0.5 s), which is consistent with the characteristics of the dosage form itself (Obaidi et al., 2013). It appears that T_max_ is quite late in some individuals (4–6 h, Figure 3) suggesting that part of the sprayed drug has been swallowed and absorbed in the gastrointestinal tract. The elimination rate constant of nasal spray is relatively small, and the t_1/2_ is prolonged. In addition, the apparent volume of distribution and clearance slightly increased. In summary, the optimized nasal spray administration method is more suitable for the test drug, which can be used as a reference for subsequent studies. In addition, we also need to pay attention to the fact that the method of administration is an important prevention and control point, which affects safety and PK/PD.

**TABLE 4:** Pharmacokinetic properties of dexmedetomidine in healthy subjects in the i.v. group or NS group.

Group	T_max_ (h)	t_1/2_ (h)	λ (1/h)	V/F (×10^5^ml)	CL/F (×10^5^ ml h^−1^)
i.v. (Part 1)	0.25 (0.28–0.22)	2.07 ± 0.33	0.34 ± 0.05	1.05 ± 0.21	0.35 ± 0.06
NS (Part 2 and Part 3)	0.5 (4.01–0.25)	3.80 ± 0.94	0.19 ± 0.05	2.54 ± 1.31	0.45 ± 0.15

Notes: Values are presented as mean ± SD, except T_max_, which is the median (min to max).

Abbreviations: PK, pharmacokinetics; NS, nasal spray; i.v., intravenous; T_max_, time to C_max_; t_1/2_, terminal elimination half-life; λ, first-order elimination rate constant; V/F, apparent volume of distribution corrected by bioavailability; CL/F, clearance corrected by bioavailability; SD, standard deviation.

The test drug did not show a significant sedative effect in Part 1. Combined with the aforementioned analysis, we believe that with the optimized administration method, Part 2 and Part 3 can truly reflect the efficacy of the test drug. The RSS of the 100-μg group increased the most and lasted the longest. The BIS scores of the 100- and 150-μg groups decreased the most, and there was no significant difference between the two doses, suggesting that 100 μg may have approached or reached the plateau of sedation. In follow-up clinical studies, choosing a dose of more than 100 μg may not increase the efficacy of the drug.

Although the test drug has not been studied in children, there is research on Dex injections in both adults and children, and the clinical application is relatively mature. Therefore, clinicians may combine the results of this study and the clinical experience of injections in adults to speculate on the doses and methods of Dex nasal spray for research or clinical application in children.

## Conclusion

In summary, Dex nasal spray with high bioavailability by the optimized administration method showed acceptable safety in a single dose of 20–100 μg and exhibited linear pharmacokinetics in healthy subjects. Dex nasal spray also showed a significant sedative effect, which presents potential clinical advantages.

## Data Availability

The original contributions presented in the study are included in the article/Supplementary Material, further inquiries can be directed to the corresponding authors.
