# Supplementary material for: Safety, Pharmacokinetics/Pharmacodynamics, and Absolute Bioavailability of Dexmedetomidine Hydrochloride Nasal Spray in Healthy Subjects: A Randomized, Parallel, Escalating Dose Study
Source: Front Pharmacol. 2022 May 20;13:871492. doi: 10.3389/fphar.2022.871492 (PMC9163677; doi:10.3389/fphar.2022.871492)
Supplement: Supplementary file 1 [file Table1.DOCX]

**Supplement**

Table S1 Pharmacokinetic properties of dexmedetomidine of healthy subjects in i.v. group or NS group.

| Group | Tmax  (h) | t_1/2_  (h) | λ  (1/h) | V/F  (×10^5^mL) | CL/F (×10^5^mL·h^-1^) |
| --- | --- | --- | --- | --- | --- |
| IV(Part 1) | 0.25(0.28-0.22) | 2.07±0.33 | 0.34±0.05 | 1.05±0.21 | 0.35±0.06 |
| NS(part 2 and part 3) | 0.5(4.01-0.25) | 3.80±0.94 | 0.19±0.05 | 2.54±1.31 | 0.45±0.15 |

Notes: Values are presented as mean ± SD, except T_max_, which is the median (min-max);

Abbreviations: PK, pharmacokinetic; NS, nasal spray; IV, intravenous; Tmax, time to Cmax; t_1/2_, terminal elimination half-life; λ, first-order elimination rate constant; V/F, apparent volume of distribution corrected by bioavailability; CL/F, clearance corrected by bioavailability; SD, standard deviatio
